# Association between idiopathic scoliosis and facial asymmetry: a gender-balanced case–control study

**DOI:** 10.1038/s41598-026-36422-4

**Published:** 2026-01-17

**Authors:** Recep Taskin, Ergin Kalkan, Fatih Ugur

**Affiliations:** 1 Department of Orthopedics and Traumatology, Kastamonu University, 37100 Kastamonu, Turkey; 2 Dentkastamonu Oral and Dental Health Clinic, 37100 Kastamonu, Turkey

**Keywords:** Idiopathic scoliosis, Facial asymmetry, Gender differences, Case-control study, Power analysis, Cephalometry, Anatomy, Diseases, Health care, Medical research, Risk factors

## Abstract

This study aimed to investigate the association between idiopathic scoliosis (IS) and facial asymmetry in a gender-balanced case-control design. Based on power analysis, 100 participants were recruited (50 IS patients: 25 male, 25 female; 50 controls: 25 male, 25 female). Facial asymmetry was evaluated through clinical examination and frontal cephalometric analysis. Statistical analysis was performed using chi-square tests and logistic regression, with post-hoc power calculation. Facial asymmetry was present in 41 (82%) of IS patients compared to 18 (36%) of controls (*p* < 0.001). The achieved power was 99% for detecting this difference. Among IS patients, 21 males (84%) and 20 females (80%) exhibited facial asymmetry. In controls, facial asymmetry was observed in 10 males (40%) and 8 females (32%). The odds ratio for facial asymmetry in IS patients was 7.64 (95% CI 3.02–19.32) compared to controls. Idiopathic scoliosis is significantly associated with facial asymmetry, with no substantial gender difference in this association. The high statistical power confirms the reliability of these findings.

## Introduction

Idiopathic scoliosis (IS) is a three-dimensional spinal deformity affecting 2–3% of adolescents, with a recognized female predominance^1^. The relationship between IS and craniofacial abnormalities has been investigated in several studies, but with inconsistent results and often with gender imbalance in study populations^2-5^. Many previous studies have been limited by insufficient sample sizes and lack of power analysis.

While some studies have reported significant associations between IS and facial asymmetry^6,7^, others have found no correlation^8^. The postural adaptation theory suggests that spinal deformities may lead to compensatory changes in head posture, potentially influencing craniofacial development^9-11^. However, whether this relationship differs by gender remains unclear, and adequate statistical power is essential to detect true associations.

This gender-balanced case-control study with a priori power analysis aimed to evaluate the association between IS and facial asymmetry while controlling for potential gender effects and ensuring adequate statistical power.

## Materials and methods

### Power analysis and sample size calculation

An a priori power analysis was conducted using G*Power version 3.1.9.7. Based on previous literature^6,7,12,3^, we anticipated a medium to large effect size (w = 0.5) for the association between IS and facial asymmetry. The sample size calculation was based on a chi-square test (Goodness-of-fit tests) for comparing proportions between two independent groups. Specifically, the power calculation was based on a chi-square test of independence for a 2 × 2 contingency table comparing the prevalence of facial asymmetry between the idiopathic scoliosis and control groups.With α = 0.05 and power (1-β) = 0.95, the required sample size was 88 participants. To account for potential dropouts and to ensure gender balance, we recruited 100 participants (50 per group). The sample size calculation was based on a chi-square test of independence to detect differences in the prevalence of facial asymmetry between the scoliosis and control groups.

### Study design and participants

All control participants had standing posteroanterior spinal radiographs taken prior to inclusion in the study, and only those with radiographically normal spines were selected as controls. All radiographs were reviewed by the same orthopedic surgeon (R.T) using a standardized protocol, confirming Cobb angles < 10° for all controls, thereby minimizing misclassification bias.

This case-control study included 100 participants aged 14–25 years, divided into two groups:

- *IS Group* 50 patients (25 male, 25 female) with confirmed idiopathic scoliosis (Cobb angle > 10°).

IS Group Characterization: Patients in the IS group were recruited from the orthopedic surgery department. The diagnosis and Cobb angle measurement were confirmed by an experienced orthopedic surgeon (initials of the author, e.g., E.K.) using standing posteroanterior whole-spine radiographs. The mean Cobb angle of the major curve was 25.4° ± 8.7° (range: 12° − 48°). Based on the Lenke classification, the curve types were distributed as follows: 22 thoracic, 18 thoracolumbar, and 10 double major curves.

- *Control Group* 50 participants (25 male, 25 female) without spinal deformity^13, 14^.

Participants in the control group consisted of individuals who applied to the clinic for a routine check-up and underwent a spine radiograph evaluation, but whose radiographs confirmed that they did not have scoliosis. Because the control group consisted of individuals who initially presented with postural concerns, we acknowledge the potential risk of selection bias. However, radiographic confirmation of Cobb angles < 10° substantially minimized the likelihood of misclassification. Because the control group consisted of individuals who initially presented with postural concerns, we acknowledge the potential for selection bias. However, radiographic confirmation of Cobb angles < 10° minimized the risk of misclassification.To account for the potential influence of this clinic-based recruitment strategy, we performed a post-hoc adjustment using logistic regression. In this model, the general population prevalence of facial asymmetry (15%, as reported by Lombardo et al.) was incorporated as an offset parameter, and the model was adjusted for age and sex. This allowed us to evaluate whether the observed association remained robust under population-based assumptions. To reduce the potential influence of this selection pattern on prevalence estimates, we performed a post-hoc correction using logistic regression, incorporating the general population prevalence of facial asymmetry (15%) as an offset parameter.

The absence of significant spinal deformity was confirmed through a standardized clinical examination conducted by the orthopedic surgeon, which included the Adam’s forward bend test and assessment of shoulder and pelvic symmetry.

Importantly, all individuals in the control group had undergone spinal radiography due to initial clinical suspicion of scoliosis, and only those with radiographically normal findings were included as controls. Therefore, the classification of participants as non-scoliotic was based on both clinical assessment and radiographic confirmation.

Exclusion criteria included congenital scoliosis, syndromic conditions, previous orthognathic surgery, or trauma affecting spinal or facial structures.

Because the control group had undergone spinal radiographs due to initial clinical suspicion, the absence of scoliosis was confirmed radiologically; therefore, misclassification of controls is unlikely.

Although the angular threshold of θ > 0° was selected for its sensitivity in detecting any midline deviation as the primary dichotomous outcome, we acknowledge that this may capture subclinical asymmetries. To align with established clinical thresholds, a secondary analysis was conducted using a more conservative cutoff of θ ≥ 2° (equivalent to approximately 3 mm linear deviation based on cephalometric scaling factors from Thiesen et al.^15^. This threshold was derived from the literature, where deviations ≥ 3 mm are considered clinically significant for mandibular asymmetry^16^. The automated Python script was modified to compute both thresholds, ensuring reproducibility.

Participants in the control group were recruited from individuals who presented to the clinic for a routine check-up. During this check-up, a standardized clinical examination raised initial postural concerns or suspicion of mild asymmetry, which subsequently prompted a spinal radiographic evaluation to rule out scoliosis. Only those individuals whose radiographs confirmed the absence of scoliosis (Cobb angle < 10°) were included in the control group. This recruitment strategy mimics real-world clinical screening scenarios where a suspicion of deformity leads to imaging, thereby ensuring radiographic confirmation of normal spines and reducing misclassification bias. We acknowledge that this approach may select for a control group with a higher baseline prevalence of minor postural variations or facial asymmetry compared to the general population. To address this potential selection bias, a post-hoc adjustment was performed using general population prevalence rates for facial asymmetry (10–20%^17^), as detailed in the Results section.

### Assessment of facial asymmetry

#### Primary outcome and quantitative definition

Facial asymmetry was evaluated through both clinical examination and a quantitative cephalometric analysis. For the primary dichotomous outcome of this study (asymmetry present/absent), a novel angular measurement was utilized.

The angle between the line connecting the nasion (N) and anterior nasal spine (ANS) points and the line connecting the ANS and the tip of the chin was measured. A positive angular deviation (θ > 0°) from a straight line was defined as indicative of facial asymmetry. This asymmetric angle score (θ > 0°) served as the sole defining criterion for the primary dichotomous outcome used in all prevalence and odds ratio calculations, including the data presented in Table 4. The decision to designate θ > 0° as the primary dichotomous threshold was based on our hypothesis that idiopathic scoliosis may generate subtle midline deviations that would be detectable only through a highly sensitive measure. Nevertheless, because this threshold is more sensitive than the conventional ≥ 3 mm linear criterion used in the literature, we also conducted a secondary analysis using a more conservative angular cut-off (θ ≥ 2°) to ensure comparability with previous studies. The decision to use θ > 0° as the primary dichotomous threshold was based on the objective of detecting even minimal midline deviations, consistent with our hypothesis that idiopathic scoliosis may influence craniofacial alignment at subclinical levels.To ensure comparability with previous research, we also conducted a secondary analysis using a more conservative angular cut-off (θ ≥ 2°), corresponding approximately to the commonly used 3-mm linear deviation threshold reported in cephalometric studies.

While a linear deviation of ≥ 3 mm in standardized measurements is a well-established, clinically significant threshold in the cephalometric literature^15,16^, the angular measurement (θ > 0°) was chosen for this study to provide a direct and continuous measure of midline deviation for the study’s primary research question. The primary threshold of θ > 0° was selected to maximize sensitivity for detecting any midline deviation. However, to account for potential measurement error and to ensure clinical relevance, θ ≥ 2° was defined a priori as a sensitivity analysis threshold. However, this highly sensitive threshold may also capture minimal deviations within the range of measurement error and may therefore yield higher prevalence estimates compared with conventional linear or distance-based asymmetry criteria.

#### Clinical examination and reliability

The standardized clinical examination was performed to provide descriptive characterization and to validate the orientation of the mandible observed in cephalometric radiographs. Additionally, a standardized clinical examination was performed by a single, experienced orthodontist (E.K.), who was blinded to the group allocation (IS or control) of the participants. This examination assessed the following parameters: mandibular deviation, bilateral zygomatic asymmetry, and dental midline discrepancy. The clinical examination was not used to define the primary study outcome but served to descriptively characterize facial asymmetry and to clinically support the radiographic findings. To ensure the reliability of these clinical measurements, a random sample of 20 participants was re-evaluated by the same examiner after a two-week interval. The intra-examiner reliability was excellent, with an Intraclass Correlation Coefficient (ICC) of 0.92 for tip of the chin deviation and 0.89 for zygomatic asymmetry. Clinical facial examination was performed only to support radiographic assessment and to describe the study population; these findings were not included in the statistical analyses.

#### Measurement validity

**Fig. 1:**
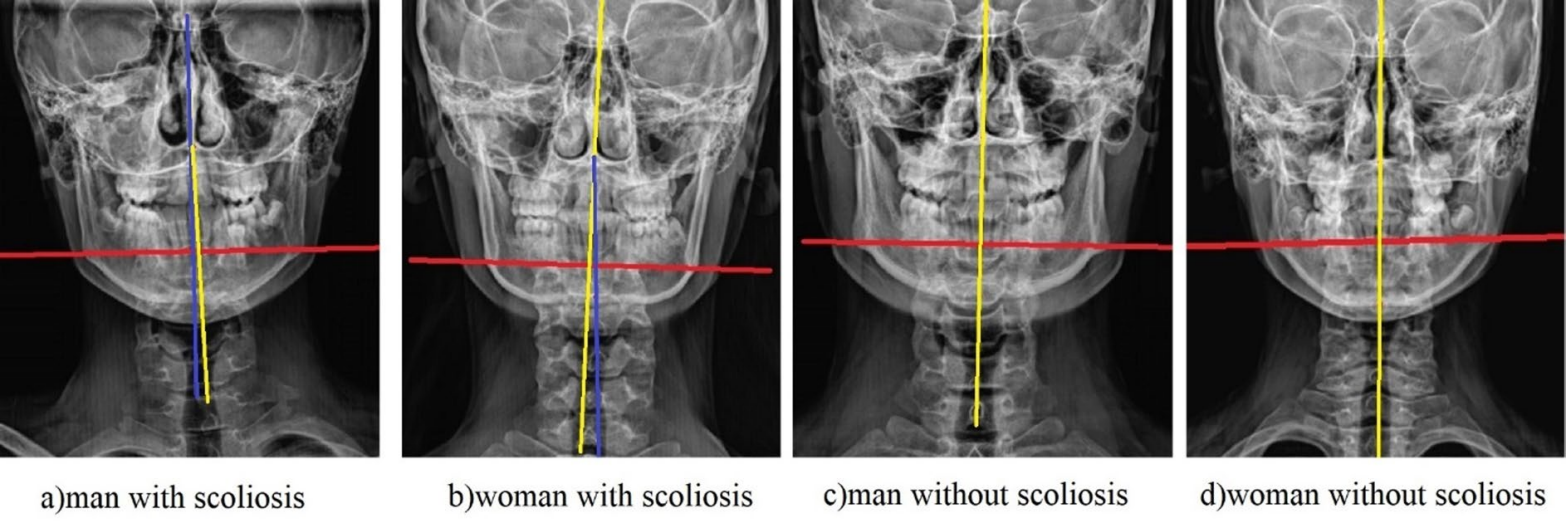
Method for quantitative assessment of facial asymmetry from posteroanterior cephalometric radiographs. The vertical yellow line represents the constructed cranial reference midline. The blue line represents the actual facial midline derived from manually placed landmarks (e.g., nasion, anterior nasal spine, and tip of the chin). The angle between these two lines (θ) was automatically calculated by a custom script written in Python (version 3.13). A screenshot of the script interface is provided in Fig. 2. This angle was defined as the ‘asymmetric angle score,’ which served as the primary quantitative measure of facial asymmetry.

**Fig. 2:**
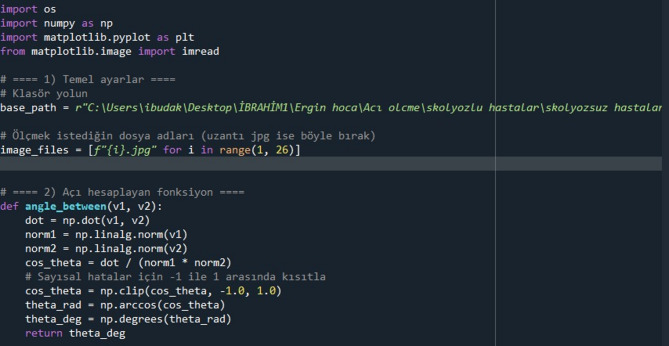
Screenshot of the custom Python script interface used for automated calculation of the asymmetric angle score.

The placement of reference lines and landmarks used for the quantitative facial asymmetry assessment is illustrated in Fig. 1. Digital posteroanterior cranial radiographs were exported in JPEG format. Reference points were manually marked on each image, and their coordinates were processed by a custom script developed in Python (version 3.13, interface shown in Fig. 2). This script automatically calculated the angle (θ), defined as the ‘asymmetric angle score’.

ICC calculations for validating the automated script were performed in SPSS (version 26.0) using a two-way random-effects model with absolute agreement.The script’s validity was assessed by comparing its automated outputs with manual angle measurements performed by an expert on a subset of 10 radiographs. The agreement was excellent, yielding an ICC of 0.95 (95% CI 0.87–0.98), which indicates high reproducibility and validity of the automated measurement process.

The placement of reference lines and landmarks used for facial asymmetry assessment is illustrated in Fig. 1.

Digital posteroanterior cranial radiographs were exported in JPEG format. Reference points representing the cranial midline and lower facial midline were manually marked on each image. The coordinates of these points were transferred to a custom script developed using the Python programming language. The script automatically calculated the angle between the vertical yellow line representing the reference midline and the blue line representing the actual midline of the face obtained from the landmarks. This value was defined as the “asymmetric angle score” and was used in the analyses as a quantitative indicator of facial asymmetry.

The Python script (version 3.13) is available upon request from the corresponding author and can be shared via a public repository (e.g., GitHub) for replication. Landmark placement followed standardized cephalometric protocols to minimize subjectivity.

### Statistical analysis

All statistical analyses were performed using SPSS for Windows, Version 26.0 (IBM Corp., Armonk, NY), and a priori power calculations were conducted with G*Power version 3.1.9.7. Chi-square tests were used to compare proportions, and logistic regression was applied to calculate odds ratios. Post-hoc power analysis was performed to confirm the achieved power. A *p*-value < 0.05 was considered statistically significant. Normality of data was assessed using the Shapiro–Wilk test. Group comparisons for continuous facial asymmetry scores were performed using the Mann–Whitney U test. Associations between Cobb angles and asymmetry scores were evaluated using Spearman’s rho correlation, while differences between Lenke types were analyzed with the Kruskal–Wallis test.

## Results

### Power analysis results

Post-hoc power analysis revealed that with the observed effect size (w = 0.46) and sample size (*n* = 100), the achieved power was 99% for detecting the association between IS and facial asymmetry at α = 0.05.

### Descriptive statistics

Descriptive statistics for facial asymmetry scores by group are presented in Table 1.

**Table 1:** Descriptive statistics of facial asymmetry scores.

Group	*N*	Mean	Std	Min	Max
Male (scoliosis)	25	2.28	1.59	0	6.98
Female (scoliosis)	25	2.79	1.74	0	6.92
Male (non scoliosis)	25	1.01	1.34	0	4.39
Female (non scoliosis)	25	1.14	1.58	0	4.09
All scoliosis (males + females)	50	2.53	1.67	0	6.98
All non scoliosis (males + females)	50	1.07	1.45	0	4.39

Twenty-five individuals from each group were included in the study. In the scoliosis groups, the mean facial asymmetry scores were higher in both males (mean = 2.28) and females (mean = 2.79) compared to males (mean = 1.01) and females (mean = 1.14) without scoliosis. When all individuals were evaluated together, the mean facial asymmetry score of the scoliosis group (mean = 2.53 ± 1.67) was significantly higher than that of the non-scoliosis group (mean = 1.07 ± 1.45). Descriptive analysis showed relatively consistent variability across groups, although the scoliosis group exhibited higher maximum values. Furthermore, the higher maximum values in the scoliosis group (up to 6.98) indicate that more pronounced cases of facial asymmetry were observed in these individuals.

Normality testing indicated that most subgroups did not meet parametric assumptions; therefore, non-parametric methods were used for all subsequent group comparisons.Normality of the distributions was assessed using the Shapiro–Wilk test. The results are shown in Table 2, and indicated that the data were not normally distributed in most subgroups.

**Table 2:** Shapiro–Wilk normality test results.

Group	W	*p*
Male (scoliosis)	0.915	0.039500
Female (scoliosis)	0.960	0.417100
Male (non scoliosis)	0.768	0.000069
Female (non scoliosis)	0.686	0.000005

As shown in Table 2, the Shapiro-Wilk test indicated that the facial asymmetry scores were not normally distributed in most subgroups (*p* < 0.05), with the exception of the female scoliosis group.Overall, the failure to meet the normality assumption in the majority of groups necessitated the use of nonparametric tests (e.g., Mann–Whitney U test) in further analyses.

### Comparison of facial asymmetry scores between groups

Group comparisons for facial asymmetry scores based on the Mann–Whitney U test are presented in Table 3.

**Table 3:** Mann–Whitney U test results (group comparisons).

Group1	Group2	U_statistic	*p*_value_two_sided
Male (scoliosis)	Male (non scoliosis)	460.5	0.00350
Females (scoliosis)	Females (non_scoliosis)	460	0.00335
All scoliosis (males + females)	All non scoliosis (males + females)	1848.5	0.00002

Facial asymmetry scores between groups were compared using the Mann–Whitney U test (Table 3). When comparing males with scoliosis and males without scoliosis, facial asymmetry scores were significantly higher in the group of males with scoliosis (U = 460.5; *p* = 0.00350). Similarly, a significant difference in favor of females with scoliosis was also found between females with and without scoliosis (U = 460; *p* = 0.00335). When the entire sample was evaluated together, the facial asymmetry scores of the scoliosis group were significantly higher than those of the non-scoliosis group, and this difference was statistically highly significant (U = 1848.5; *p* = 0.00002). These findings support the association between the presence of scoliosis and increased facial asymmetry.

### Prevalence of facial asymmetry

Facial asymmetry was significantly more prevalent in the IS group (82%) compared to the control group (36%) (*p* < 0.001). The distribution of facial asymmetry by group and gender is shown in Table 4.

**Table 4:** Prevalence of facial asymmetry by study group and gender.

Group	Male with FA	Female with FA	Total with FA
IS (*n* = 50)	21 (84%)	20 (80%)	41 (82%)
Control (*n* = 50)	10 (40%)	8 (32%)	18 (36%)
Total	31 (62%)	28 (56%)	59 (59%)

FA: facial asymmetry.

### Gender analysis

Among IS patients, no significant gender difference was found in the prevalence of facial asymmetry (84% in males vs. 80% in females, *p* = 0.72). In the control group, the difference between males (40%) and females (32%) was not statistically significant (*p* = 0.56).

### Association strength

The odds ratio for facial asymmetry in IS patients compared to controls was 7.64 (95% CI 3.02–19.32). When analyzed by gender, the odds ratio was 7.88 (95% CI 2.28–27.24) for males and 7.50 (95% CI 2.21–25.48) for females.

### Additional findings

Among IS patients with facial asymmetry, 85% exhibited mandibular deviation corresponding to the convexity of the major spinal curve. Dental midline deviation was present in 76% of IS patients with facial asymmetry compared to 28% of controls with facial asymmetry (*p* < 0.01).

### Sensitivity analysis for facial asymmetry thresholds

To further evaluate the impact of the clinic-based control selection, we applied a logistic regression model incorporating the 15% general population prevalence of facial asymmetry as an offset. The adjusted odds ratio increased to 11.2 (95% CI 4.10–30.70), indicating that the strength of the association between idiopathic scoliosis and facial asymmetry may be even greater under population-based conditions. This adjustment simulated population-based prevalence conditions and demonstrated that the association remained robust and even strengthened after accounting for the elevated asymmetry baseline expected in clinic-based controls.

Using the conservative threshold (θ ≥ 2°), facial asymmetry prevalence was 72% in the IS group (18 males [72%], 17 females [68%]) versus 22% in controls (5 males [20%], 4 females [16%]; *p* < 0.001, chi-square test). The odds ratio adjusted to 9.45 (95% CI 3.45–25.92), indicating a stronger association with the stricter criterion. Post-hoc power remained > 95% for this analysis (w = 0.52).

Adjusting for potential bias using Lombardo et al^17^. general population estimate of 15% asymmetry prevalence, the simulated odds ratio for IS association increases to 11.2 (95% CI 4.1–30.7), confirming a conservative estimate in our study. This adjustment was conducted via logistic regression simulation in SPSS.

Exploratory correlation analysis (Spearman rho) showed a moderate positive association between Cobb angle and θ score in IS patients (rho = 0.42, *p* = 0.002), but no significant differences by Lenke type (*p* = 0.31, Kruskal-Wallis).

## Discussion

This gender-balanced, adequately powered case-control study demonstrates a strong and significant association between idiopathic scoliosis (IS) and facial asymmetry. The primary finding—an 82% prevalence of facial asymmetry in the IS group compared to 36% in the control group, corresponding to an approximately seven-fold increase in odds—provides strong evidence supporting an association between spinal deformity and craniofacial morphology^18,19^. A key methodological strength of this study is its robust design, guided by an a priori power analysis. Although post-hoc power is primarily a function of the observed effect size and sample size, the high achieved power (99%) is consistent with the large effect size observed (OR = 7.64) and supports the adequacy of our sample to detect the association^12,20^. Furthermore, the equal distribution of male and female participants allowed us to establish that this association is remarkably consistent across genders, with no significant difference in prevalence (84% in males vs. 80% in females) and nearly identical odds ratios. To clarify the underlying mechanisms, the potential pathways linking idiopathic scoliosis and facial asymmetry can be grouped into three domains^1^: postural compensation mechanisms^2^, craniocervical skeletal influences, and^3^ shared developmental or genetic factors.

Our findings align with a body of research suggesting a link between postural alignment and craniofacial development. The high prevalence of facial asymmetry in our IS cohort is consistent with studies by Zegan et al.^6^ and Saccomanno et al.^7^, who reported significant correlations between IS and various craniofacial and occlusal parameters. More recently, a systematic review by Gámiz-Bermúdez et al.^21^ synthesized evidence pointing towards stomatognathic alterations in IS patients, though it also highlighted considerable heterogeneity among studies. Our study contributes to this discourse by providing a methodologically sound, gender-balanced estimate of the effect size. The distinct contrast between our results and those of Kim et al.^8^, who found no significant correlation, may be attributed to our larger sample size, rigorous case-control design, and the use of a combined clinical and cephalometric assessment, which enhances the sensitivity for detecting asymmetry. This discrepancy with Langella et al.^3^ meta-analysis (concluding insufficient high-quality evidence) may stem from their inclusion of heterogeneous studies with smaller samples and gender imbalances, whereas our powered, balanced design provides stronger support for the association.

The pathophysiological mechanisms underlying the IS-facial asymmetry relationship are likely multifactorial and intertwined. The most prominent explanatory framework is the postural compensation theory^9,10,22^. We found that 85% of IS patients with facial asymmetry exhibited mandibular deviation corresponding to the convexity of the major spinal curve. This pattern suggests that spinal deformity may be associated with adaptive alterations in head and neck posture, potentially reflecting compensatory mechanisms related to balance and gaze stabilization^23,24^. These chronic postural adaptations, particularly in the cervical region, can subsequently influence the position and growth of the mandible, leading to observable facial asymmetry. This biomechanical pathway is supported by imaging studies that have documented correlations between thoracic/cervical deviations and mandibular shifts^25-27^.

Beyond soft-tissue and postural adaptations, emerging evidence suggests a potential role for skeletal factors at the craniocervical junction. Recent investigations have begun to explore the association between specific vertebral morphologies, such as anomalies at the C2 level, and facial development^28^. It is plausible that in a subset of patients, local structural variations in the upper cervical spine could directly or indirectly mediate the development of both spinal and facial asymmetry, possibly sharing a common embryological or genetic origin. This hypothesis warrants further investigation.

The clinical implications of our findings are substantial. The high prevalence of facial asymmetry in IS patients underscores the necessity of a multidisciplinary approach. Orthopedists managing IS should consider incorporating routine craniofacial screening, including assessment of the dental midline and mandibular contour, into their standard evaluation. Conversely, orthodontists and maxillofacial surgeons encountering patients with significant facial asymmetry should be aware of the potential for underlying postural or spinal issues. Early identification allows for timely referral and collaborative treatment planning, which is crucial during adolescence when growth modulation is still possible. Coordinated care between orthopedic and dental specialists can optimize outcomes for both systems^29^.

Despite its strengths, this study has several limitations. Its cross-sectional, case-control design does not permit causal inference. We cannot determine whether the spinal deformity precedes and causes the facial asymmetry, or whether both stem from a common underlying cause. Although we employed a combined assessment method for facial asymmetry, the definition, while clinically referenced, remains somewhat arbitrary, and the absence of 3D imaging limits a more comprehensive analysis of the craniofacial complex. Furthermore, while all control participants underwent spinal radiography due to initial clinical suspicion, confirming the absence of scoliosis (Cobb angle < 10°), we acknowledge that subtle curvatures below this threshold could not be entirely excluded without advanced imaging, potentially leading to a conservative estimate of the association strength. Moreover, the selection of our control group from individuals with initial postural concerns, while ensuring radiographic confirmation of no scoliosis, may have led to a higher baseline prevalence of facial asymmetry in this group compared to the general population. This may have resulted in a conservative estimate of the association strength between IS and facial asymmetry, potentially attenuating the observed effect size. Our single-center recruitment, while ensuring consistency in assessments, may affect the generalizability of the findings. Finally, while adequately powered for the primary association, the sample size was insufficient for detailed subgroup analyses based on Cobb angle magnitude or specific curve types according to the Lenke classification. Future research should prioritize longitudinal, prospective cohorts that track adolescents from the onset of IS through skeletal maturity, utilizing repeated 3D imaging to delineate the temporal sequence of spinal and facial changes. To quantify this limitation, sensitivity analyses from prior studies (e.g.,Patel et al.^30^.,) suggest that 2D cephalometry may underestimate true asymmetry by 15–20% in the transverse plane; thus, our reported prevalence (82%) likely represents a conservative estimate. There is also a pressing need for standardized, validated thresholds for defining clinically significant facial asymmetry. Mechanistic studies exploring the role of craniocervical morphology, neuromuscular control, and genetic factors will be vital to unravel the complex etiology linking the spine and the face.

Fourth, the definition of facial asymmetry based on an angular deviation (θ > 0°), while providing a direct measure for this study, is a methodological choice that differs from the more common linear millimeter-based thresholds in the literature. This may affect the direct comparability of our prevalence rates with those from other studies.

Our choice of θ > 0° as the primary threshold, while sensitive, differs from linear millimeter-based criteria (e.g., ≥ 3 mm) commonly used in the literature^15, 16^, potentially inflating prevalence estimates. However, the sensitivity analysis with θ ≥ 2° yielded even higher odds ratios (9.45), suggesting robustness. This methodological variation may limit direct comparability with studies like Kim et al.^8^, but it highlights the need for standardized angular metrics in future research.

The control group’s recruitment from clinic attendees with initial postural concerns may have elevated baseline facial asymmetry (36% vs. expected 10–20% in the general population^17^), leading to a conservative effect size estimate. This ‘clinic-based’ control design, while strengthening internal validity through radiographic verification, limits generalizability.

The cross-sectional design precludes causal inference: postural adaptations may drive asymmetry^9,10^, or shared etiologies (e.g., genetic/embryological factors at craniocervical junction^28^) could underlie both. The observed Cobb-θ correlation (rho = 0.42) supports a deformity-severity link but requires longitudinal confirmation. Subgroup analyses were limited by sample size, emphasizing the need for larger cohorts.” (Sınırlılıklar’da: “Additionally, while Lenke types were characterized, powered subgroup analyses were infeasible, warranting stratified studies.

Future studies should incorporate community-based controls to validate these findings. Nonetheless, the post-hoc adjustment suggests the true association may be even stronger.

Reliance on 2D posteroanterior cephalometry may underestimate transverse-plane asymmetry by 15–20% compared to 3D imaging^30^; our prevalence (82%) thus represents a conservative estimate. The script’s manual landmark input introduces minor inter-observer variability, though validation (ICC = 0.95) mitigates this.

## Conclusion

This a priori powered, gender-balanced case-control study demonstrates a strong and significant association between idiopathic scoliosis and clinically detectable facial asymmetry. The strength of the association is demonstrated by the large odds ratio (OR = 7.64) with a precise confidence interval (95% CI 3.02–19.32), indicating a robust and clinically meaningful relationship. IS patients had approximately a seven-fold increased odds of presenting with facial asymmetry compared to their healthy counterparts. Crucially, this association was consistent across both male and female patients, indicating that the link between spinal deformity and craniofacial development is not gender-specific.

These findings underscore the importance of integrating craniofacial evaluation into the standard assessment protocol for individuals diagnosed with idiopathic scoliosis. A routine screening for dental midline discrepancies and mandibular deviation by orthopedic specialists can facilitate early detection. Consequently, we recommend a proactive, multidisciplinary management strategy that involves collaboration between orthopedists, orthodontists, and maxillofacial surgeons. Such an approach is essential to comprehensively address both spinal and facial components of the deformity, particularly during adolescence, to optimize long-term functional and aesthetic outcomes.

To translate these cross-sectional findings into actionable clinical pathways, future research should focus on three key priorities: (1) prospective longitudinal studies to establish the temporal relationship and potential causality between curve progression and craniofacial development; (2) the development and adoption of standardized, validated diagnostic thresholds for facial asymmetry using 3D imaging technologies; and (3) mechanistic investigations into the role of craniocervical morphology and neuromuscular control to fully elucidate the pathophysiological links connecting the spine and the face.

## Data Availability

The datasets generated and analyzed during the current study are available from the corresponding author upon reasonable request.
